# Addictive Behaviors among University Students in Kenitra, of Internet and Social Media: An Exploratory Study

**DOI:** 10.1192/j.eurpsy.2025.938

**Published:** 2025-08-26

**Authors:** A. Chaker Lamrani, S. Boulbaroud, M. Rmili, M. Alayachi, F.-Z. Azzaoui

**Affiliations:** 1 Ibn Tofail University, Laboratory of Biology and Health, KENITRA; 2 Sultan My. Slimane University, Polydisciplinary Faculty, BeniMellal, Morocco

## Abstract

**Introduction:**

As the accessibility of new technology becomes easier and its usage longer, especially among university students, the repercussions on content consumption and behaviors must be examined and addressed, both for prevention and remediation.

**Objectives:**

The aim of this study is to assess the degree of addictive behaviors associated with the use of the internet and social media. Through a questionnaire, data was collected from a random sample of university students in Kenitra.

**Methods:**

58 students participated in the study (21.29 ± 1.69) (38 women and 20 men), distributed as follows: 31% in the first year, 32% in the second year, and 36% in the third year. Of these, 94% reported owning a smartphone for at least one year.

**Results:**

17% reported using internet for more than 12 hours per day. 37% recharge their balance immediately after it is depleted, and 41% become frustrated when they receive no response to a message, 38% are constantly awaiting new notifications. Nearly 30% reported that internet use affects their studies, health, and causes conflicts with their parents.

Girls are more connected than boys and cannot tolerate being without internet access (p < 0.05, respectively). The number of repeated semesters is highly correlated with the duration of smartphone ownership and significantly correlated with age (p < 0.05). Furthermore, a strong correlation is observed between the duration of internet use and the allocated budget (p < 0.05), as well as the ability to tolerate the absence of internet access (p < 0.05).

The regression test shows that an increase in the allocated budget for internet connection leads to an increase in the time spent using smartphone applications (β = 0.42), and the model explains 47% of this relationship. Additionally, the nature of the application used can explain 30% of the increase in the number of repeated semesters among university students (β = 0.30), this parameter is also explained by the duration of smartphone ownership by 10% (β = 0.33).

**Conclusions:**

Internet access is facilitated by smartphones, which fosters attachment behaviors among university students that can lead to addiction and certainly have an influence on academic progress, health, and family relationships.

**Disclosure of Interest:**

None Declared

